# Isolate and irreducible radial head dislocation in children: a rare case of capsular interposition

**DOI:** 10.1186/s12891-020-03685-5

**Published:** 2020-10-07

**Authors:** Luigi Tarallo, Michele Novi, Giuseppe Porcellini, Fabio Catani

**Affiliations:** Orthopedics and Traumatology Department, University of Modena and Reggio Emilia, Policlinico di Modena, Modena Via del Pozzo 71, 41124 Modena, Italy

**Keywords:** Locked radial head dislocation, Children elbow dislocation, Capsular button-holed

## Abstract

**Background:**

Radial head dislocation with no associated lesions, is a relatively uncommon injury in children.

In this case report, it is reported a case of anteromedial locked radial head dislocation in children, and we discuss its clinical presentation and pathogenetic mechanism of injury.

**Case presentation:**

An 8-year-old girl fell off on her right forearm with her right elbow extended in hyperpronation. An isolated radio-capitellar dislocation was identified with no other fractures or neurovascular injuries associated. Elbow presented an extension-flexion arc limited (0°- 90°), and the prono-supination during general anesthesia shows “a sling effect” from maximal pronation (+ 55°) and supination (+ 90°) to neutral position of forearm. The radial head dislocation was impossible to reduce and an open reduction was performed using lateral Kocher approach. The radial head was found “button-holed” through the anterior capsule. The lateral soft tissues were severely disrupted and the annular ligament was not identifiable. Only by cutting the lateral bundle of the capsule was possible to reduce the joint.

At 50 moths follow-up, patient presented a complete Range of motion (ROM), complete functionality and no discomfort or instability even during sport activities.

**Discussion and conclusion:**

It is important to understand the pathogenic mechanisms of locked radial head dislocation in children. Some mechanism described are the distal biceps tendon or the brachialis tendon interposition. However even the anterior capsule can hinder reduction. A characteristic “sling-effect” of the forearm could be pathognomonic for capsular button-holing. Surgical release of the capsular bundle sometimes is the only way to reduce the dislocation and obtain a good outcome.

## Background

Radial head dislocation rarely occurs in patients younger than 8 years old, (peak incidence of elbow dislocation is 12–13 years of age) and it is often associated with ulna fracture such as a Monteggia injury or plastic deformation injuries described as Monteggia-variant [1, 2]. The radial head subluxation (“pulled elbow” or “nursemaid elbow”) however is a common traumatic condition in pediatric population, that mainly occurs in children between 1 and 4 years of age and it can generally be reduced with forced flexion of the elbow associated with full supination. If the anterolateral radial head dislocation is common, on the other hand a true radio-capitellar dislocation is a relatively uncommon injury in children. Often, anterior radio-capitellar dislocations may occur as the result of combined axial, torsional, and/or extension forces, and associated injury such as fracture, ulno-humeral dislocation, and ligamentous disruption [3]. Usually the commonly noted block to anatomic reduction of radial head dislocation is an interposed annular ligament which, if damaged, should be repaired to maintain stable joint congruency following reduction [4, 5]. In this case report, we present a case of anteromedial radial head dislocation in children, and discuss its clinical presentation and pathogenetic mechanism of injury.

## Case presentation

**Fig. 1:**
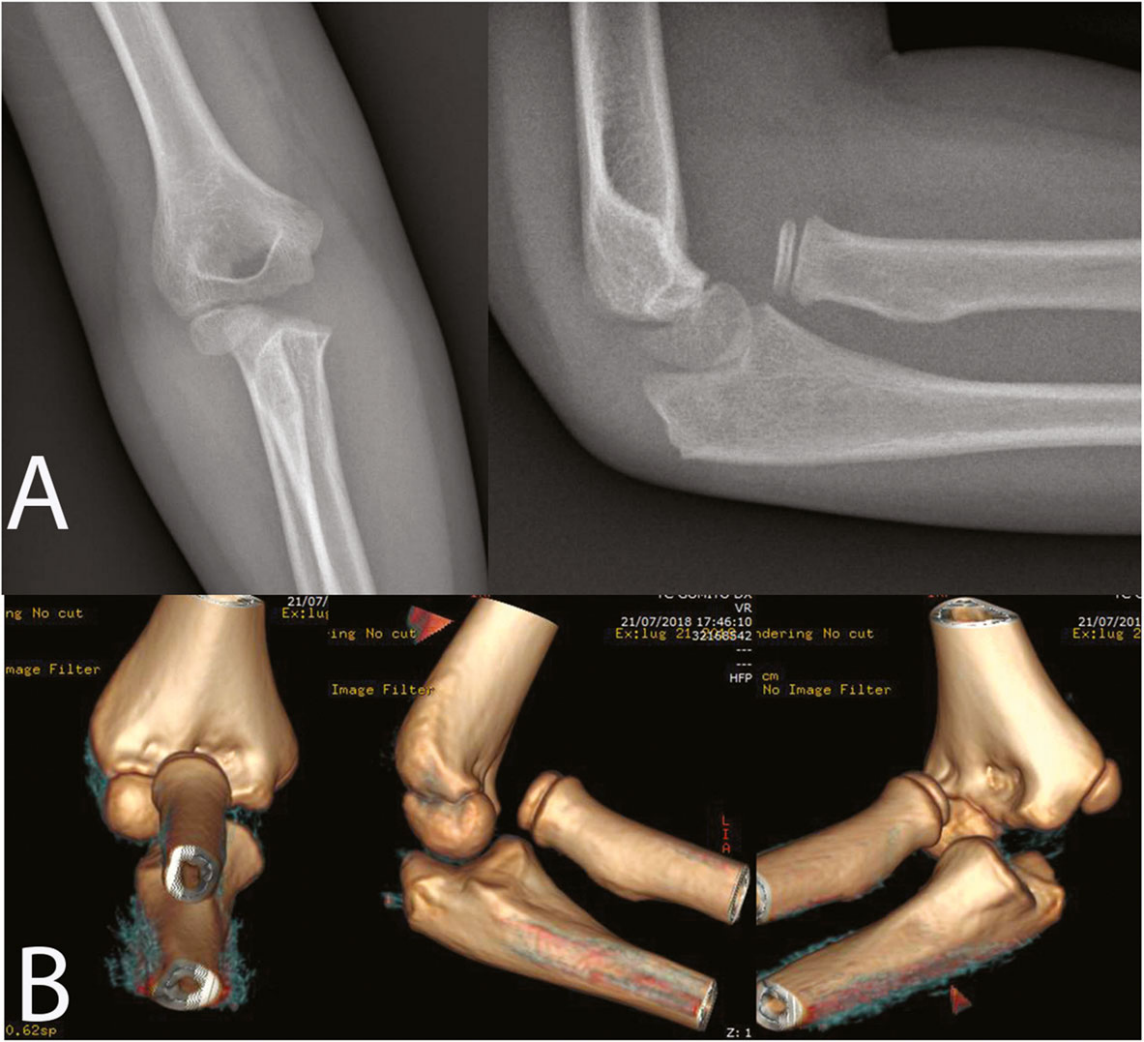
X-ray (**a**) and CT scan (**b**) of radial head dislocation

**Fig. 2:**
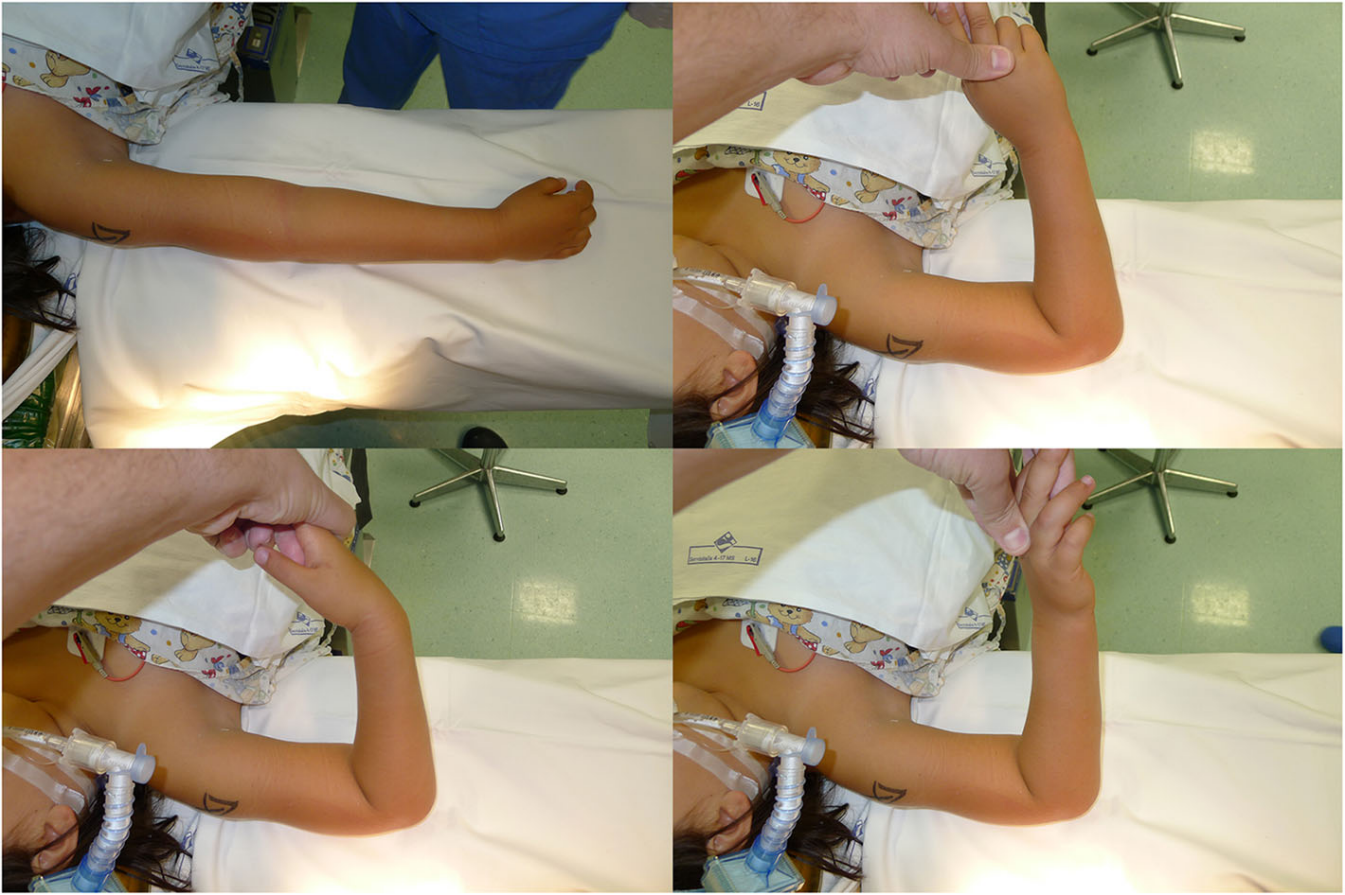
Pre-Operative clinical examination

**Fig. 3:**
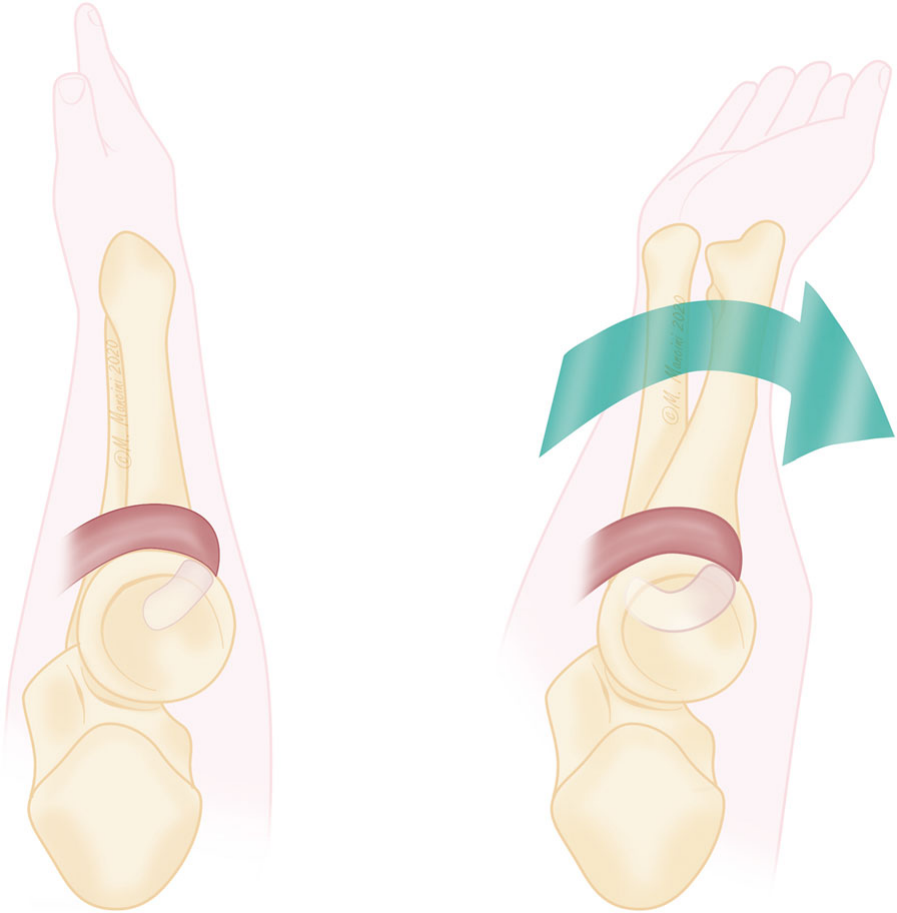
The “sling effect” from the maximal supination to neutral position of the forearm, due to the capsular interposition

**Fig. 4:**
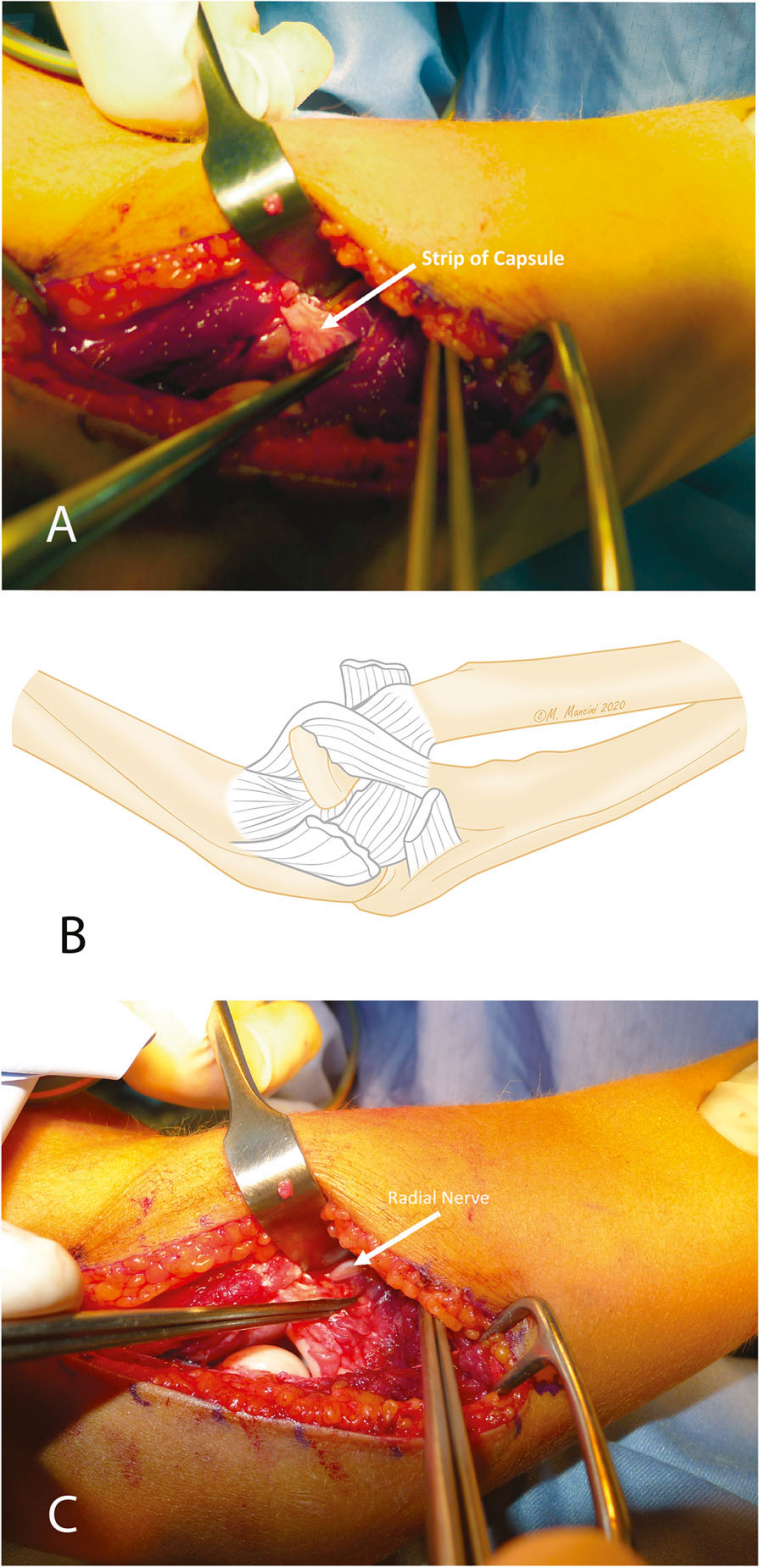
Radial head dislocation with button-holing of the capsule (**a**); Pathogenic mechanism illustration (**b**); Reduction of the dislocation and radial nerve identification (**c**)


An 8-year-old girl fell off a table, landed on the ground, and twisted her right forearm with her right elbow extended in hyperpronation. Clinical presentation of the elbow was swelling, pain and a severe functional limitation, but there were no signs of neurologic or vascular injury. X-ray, and a CT scan was carried out to identify any associated fractures. An isolated radio-capitellar dislocation was identified at the emergency room where an unsuccessful closed reduction attempt was carried out (Fig. 1a-b). The day after the trauma, on examination under anesthesia, forearm rotation and flexion-extension were examined (Fig. 2). The patient presented a limited extension-flexion arc of motion (0°- 90°) and the prono-supination during general anesthesia showed “a sling effect” with an elastic return from maximal pronation (+ 55°) and maximal supination (+ 90°) to neutral position of forearm (Fig. 3 and video attached in supplementary contents). The radial head dislocation was impossible to reduce with the elbow fully flexed and supinated; no elbow instability to varus or valgus stress was assessed under fluoroscopic examination. After attempting an unsuccessful closed reduction of the radial head subluxation under general anesthesia, an open reduction was carried out using postero-lateral Kocher approach. The surgical exposure of the joint using Kocher approach, revealed an anterior dislocation of the radial head button-holed through the anterior capsule (Fig. 4a). The lateral soft tissues were severely disrupted and the annular ligament was not identifiable. The radial head was dislocated anteriorly, with elbow in extension and pushed anteromedial by the lateral border of the joint capsule (Fig. 4b). Only by cutting a bundle of the capsule without violating the annular and collateral ligament was it possible to reduce the radial head in the anatomical position, with accurate blunt dissection the radial nerve was identified (Fig. 4c) and a complete flexion-extension arc of motion was obtained; 80° of pronation and 85° of supination have been reached. The elbow was stable throughout the entire range of movement. The patient’s arm was set in a plaster cast and immobilized in 90° of flexion for 3 weeks. Postoperatively, radiographs demonstrated anatomic radio-capitellar alignment. Six months post-injury, her right elbow showed a complete (ROM), no residual instability and the X-rays also showed an anatomical congruence of radio-capitellar joint.

**Additional file 1.**

At 50 moths followup, patient presented a complete ROM, complete functionality and no discomfort during sport activities.

## Discussion and conclusion

**Fig. 5:**
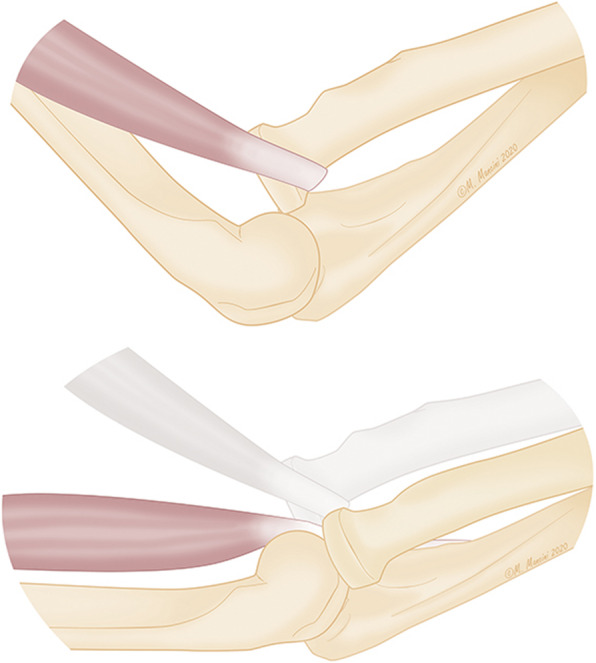
Mechanism of disengagement of the radial head from the brachial tendon

Traumatic radial head dislocation is usually associated with fractures of the forearm [6, 7]. Isolated radial head dislocation in children is rare and most of this injury represent a monteggia-variant lesion, with plastic deformation of the ulna. In most cases of acute traumatic radial head dislocation, closed reduction is easily accomplished. Failure of closed reduction is often due to soft tissue interposition with either the annular ligament or joint capsule [8]. C. Camp and O’Driscoll, described a buttonholing of the radial head in the brachialis as a cause for irreducible dislocation including an anterior and medially dislocated radial head in absence of identifiable intra-articular block of joint reduction [3]. This pathogenetic mechanism described by Camp in the anatomic dissection, shows an elbow in flexion of 90° and cannot clarify the complete extension of the elbow allowed in our case. Furthermore, an extension of the elbow could lead to disengage the radial head from the lateral portion of brachial tendon during the extension maneuver (Fig. 5) [9]. In our case the radial head seems locked in a sort of buttonholing on the anterior capsule and pushed medially with respect to the anatomical position. The clinical presentation of the patient showed an arc of movement of complete extension and limited flexion to 90°, associated with an elastic return to neutral position either from maximal pronation and maximal supination, that we described as a “sling effect”. If a loop of the biceps tendon around the radial neck was present, as described by Vidyadhar V. Upasani [10], it can only explain this elastic return when the forearm pass from supination to neutral position like a sort of “spring preloading” but not in the opposite side from maximal pronation to neutral position because the tendon is released. In our case we found this sling-effect both in supination and pronation.

In our case, we described a mechanism very similar to brachialis tendon interposition described by Camp, in fact the CT scan images are very similar, but we found a strip of the capsule which locked the radial head in the volar and medial position without any possibility of reducing the dislocation of radial head. All attempts to reduce the radial head dislocation during surgery were superfluous.

The capsule, interposed between the radial head and the capitellum, blocked reduction of the radial head and had to be incised to restore joint alignment. In conclusion we suggest considering the buttonholing of the radial head incarcerated on the volar capsule in presence of irreducibility of the radial head, if the patient presents an arc of movement of 0–90°, a supination relatively complete 90° and a limited pronation. Another interesting sign is the clinical presentation of the forearm that passes from complete supination to neutral position in a sort of “sling” as well as from maximal pronation to neutral position.

## Data Availability

Data that support the findings of the study are available from the corresponding author on reasonable request.

## Abbreviation

ROM: Range of motion
